# Surgical resection of pulmonary crystal-storing histiocytosis with Sjögren's syndrome: A case report

**DOI:** 10.1016/j.ijscr.2021.106196

**Published:** 2021-07-14

**Authors:** Soichiro Kiya, Shigeyuki Morino, Keisuke Iwasaki, Akihiro Nakamura

**Affiliations:** a Department of Chest Surgery, Sasebo City General Hospital, 9-3 Hirase-cho, Sasebo, Nagasaki 857-8511, Japan; b Department of Pathology, Sasebo City General Hospital, 9-3 Hirase-cho, Sasebo, Nagasaki 857-8511, Japan

**Keywords:** CSH, crystal-storing histiocytosis, LP-PCD, lymphoproliferative or plasma cell disorder, MM, multiple myeloma, LPL, lymphoplasmacytic lymphoma, MGUS, monoclonal gammopathy of undetermined significance, CT, computed tomography, FDG, fluorodeoxyglucose, PET, positron emission tomography, Ig, immunoglobulin, Pulmonary crystal-storing histiocytosis (CSH), Sjögren's syndrome, Surgical resection, Case report

## Abstract

**Introduction and importance:**

Crystal-storing histiocytosis (CSH) is a rare clinical entity characterized by an abnormal increase in the number of histiocytes with massive accumulation of crystallized immunoglobulins. Yano et al. reported only one case of gastric CSH associated with Sjögren's syndrome. In this report, we present a case of pulmonary CSH with Sjögren's syndrome, and discuss the relevant literature.

**Case presentation:**

A 64-year-old woman who had never smoked presented with cough 2 years earlier. Chest CT showed that the nodule in the right lower lobe had slowly enlarged to 12 × 10 mm. We suspected primary lung cancer and performed video-assisted thoracoscopic right S6 segmentectomy. Histopathological evaluation of the resected specimen revealed crystal-storing histiocytosis. As of 6 months postoperatively, no recurrence has been identified.

**Clinical discussion:**

Eighteen cases of pulmonary CSH have been described in the English language peer-reviewed literature, including our case. In this case, the patient had a history of Sjögren's syndrome, but no lymphoproliferative or plasma cell disorder (LP-PCD). Therapy for all patients without LP-PCD was excisional resection of the lung. Treatment and prognosis of patients with CSH varied according to the defined pathology. Jones et al. reported the case of 54-year-old woman without LP-PCD who presented with a solitary asymptomatic focus of CSH in the lung and initially underwent lesion resection, but showed recurrence 10 years later.

**Conclusion:**

Pulmonary CSH is one differential diagnosis for pulmonary nodule enlargement in patients with autoimmune disease. Surgical resection appears to represent an effective therapeutic option for localized CSH, but long-term follow-up remains necessary.

## Highlights

• Pulmonary crystal-storing histiocytosis (CSH) with Sjögren's syndrome is extremely rare.
• Pulmonary CSH is one differential diagnosis for pulmonary nodule enlargement in patients with autoimmune disease.
• Surgical resection appears to represent an effective therapeutic option for localized CSH.
• Although pulmonary CSH is a benign tumor, long-term follow-up is warranted.

## 1 Introduction

Crystal-storing histiocytosis (CSH) is a rare clinical entity characterized by an abnormal increase in the number of histiocytes with massive accumulation of crystallized immunoglobulins. About 90% of CSH cases have an underlying lymphoproliferative or plasma cell disorder (LP-PCD) such as multiple myeloma (MM), lymphoplasmacytic lymphoma (LPL), or monoclonal gammopathy of undetermined significance (MGUS). The remaining 10% of CSH cases are associated with autoimmune diseases, reactive inflammatory processes secondary to infections or other diseases (e.g.: Crohn disease), metabolic disorders, or drugs (e.g.: clofazimine) [1]. Yano et al. reported only one case of gastric CSH associated with Sjögren's syndrome [2].

In this report, we present a case of pulmonary CSH with Sjögren's syndrome, and discuss the relevant literature. This case report has been reported in line with the SCARE Criteria [3].

## 2 Case presentation

**Fig. 1:**
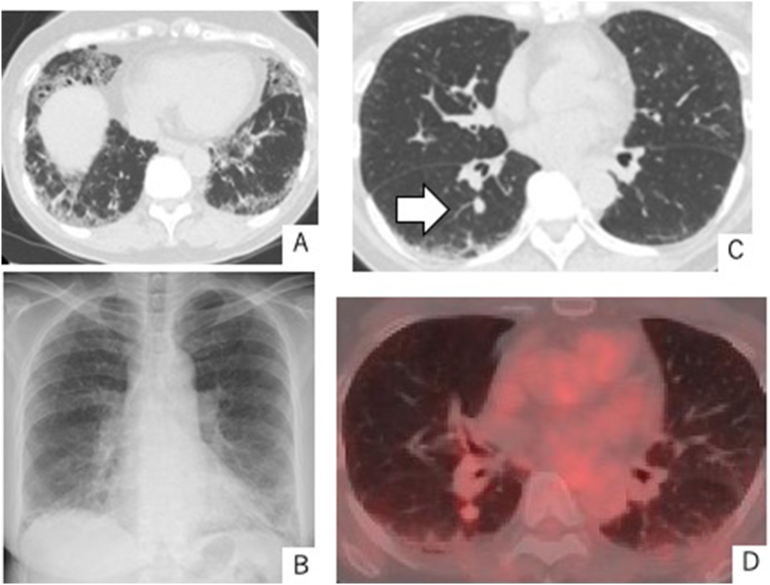
A: Chest CT reveals interstitial shadows, predominantly in bilateral lung bases. B: Chest radiography reveals no nodules. C: Chest CT reveals a 12 × 10 mm nodule (arrow) in the S6 segment of the right lower lobe. D: Accumulation of ^18^F-FDG is apparent in the area of nodular shadows on PET/CT.

**Fig. 2:**
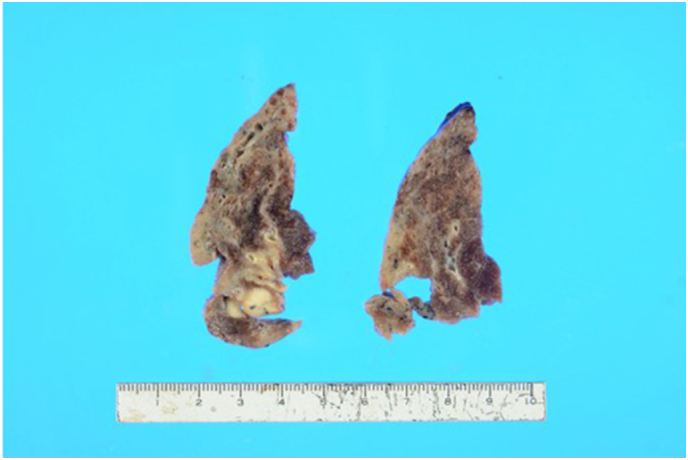
Macroimage of the resected specimen showing the solitary nodule (8 × 8 mm).

A 64-year-old woman who had never smoked presented with cough 2 years earlier. She had past medical history of asthma, and surgical history of appendectomy. She had no history of drug consumption. There was no family history associated with this disease. Computed tomography (CT) of the chest revealed a 10 × 8 mm nodule in the S6 segment of the right lower lobe. In addition, interstitial shadows were found predominantly in the bilateral lung bases (Fig. 1A). A systematic examination for interstitial shadows revealed positive anti-SS-A antibodies and hyposecretion of saliva, leading to the diagnosis of Sjögren's syndrome. Two years later, chest radiography revealed no nodule (Fig. 1B), whereas chest CT showed that the nodule in the right lower lobe had slowly enlarged to 12 × 10 mm (Fig. 1C). Transbronchial lung biopsy was not performed because of the difficult location of the nodule. Tumor markers (CEA, CYFRA, pro-GRP) were not elevated. Cryptococcal antigen was negative. Accumulation of ^18^F-fluorodeoxyglucose (FDG) was observed in the nodular shadows (Fig. 1D), but no significant accumulation of ^18^F-FDG in hilar or mediastinal lymph nodes was seen on positron emission tomography (PET)/CT. We suspected primary lung cancer and performed video-assisted thoracoscopic right S6 segmentectomy. A macroimage of the resected specimen showed a solitary nodule (8 × 8 mm) (Fig. 2). The nodule was soft to the touch. The lung tissue around the specimen showed fibrosis of the subpleural stroma, focal infiltration of lymphocytes, honeycombing of the glandular epithelium, small cyst formation, and bronchiectasis.

**Fig. 3:**
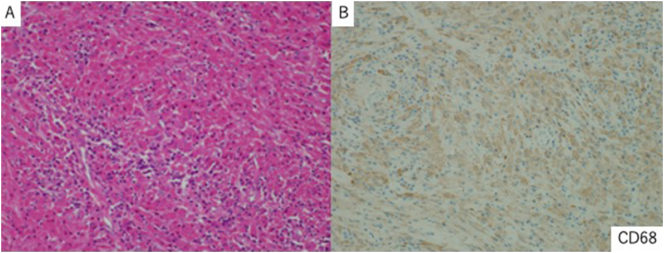
A: The tumor area shows infiltration of lymphocytes and plasma cells, and a solitary increase in the number of histiocytes with abundant eosinophilic cytoplasm and unevenly distributed small nuclei, and fibrotic foci with vitrification in the center (×20). B: Immunostaining of the resected specimen reveals that the crystal-storing histiocytes are positive for CD68.

**Fig. 4:**
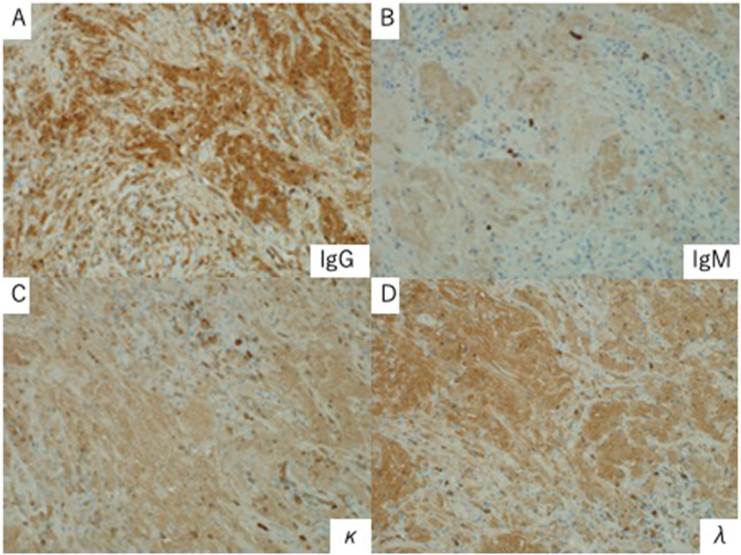
Immunostaining of the crystal-storing histiocytes reveals positive results for IgG (A), weakly positive results for IgM (B), and positive results for κ (C) and λ chains (D).

The tumor area showed infiltration of lymphocytes and plasma cells, and a solitary increase in the number of histiocytes (CD68+) with abundant eosinophilic cytoplasm and unevenly distributed small nuclei, and fibrotic foci with central vitrification (Fig. 3). Immunostaining showed CD68 (+), S-100 (−), κ (+), λ (+), immunoglobulin (Ig) G (+), IgM (weak+), and IgA (−) (Fig. 4). Histopathological evaluation of the resected specimen revealed crystal-storing histiocytosis. The postoperative course was uneventful, and the patient was discharged on postoperative day 7. As of 6 months postoperatively, no recurrence has been identified.

## 3 Discussion

CSH is broadly classified into generalized and localized types. Generalized CSH is defined as the involvement of two or more distant organs or sites. In a review of 80 patients with CSH by Dogan et al. [1], the most commonly affected sites were the bone marrow (97%), liver (47%), lymph nodes (44%), spleen (44%), and kidney (38%). The lung was involved in only about 10% of cases. Localized CSH was defined as a single deposition involving only one organ or site. The most common site was the head and neck, followed by the lungs. Our case was classified as pulmonary localized CSH.

In our case, the nodule in the S6 segment of the right lower lobe tended to increase in size and showed accumulation on PET/CT. Wedge resection of the lung and submission for rapid histology would have been preferable. If malignant findings had been present, right lower lobectomy should have been performed, but the location of the tumor made wedge resection difficult. The patient had interstitial lung disease associated with Sjögren's syndrome and was thus at risk of acute exacerbation of interstitial pneumonia after pulmonary resection. Considering the risk of acute exacerbation of interstitial pneumonia, we chose right S6 segmentectomy instead of right lower lobectomy to reduce the lung volume to be resected.

Although pulmonary CSH was difficult to differentiate from lung cancer preoperatively, histopathological examination revealed not lung cancer, but pulmonary crystal-storing histiocytosis. The tumor area showed infiltration of lymphocytes and plasma cells. The plasma cells included more κ-positive cells than λ-positive cells, but the monoclonality was not clear. The patient had a history of Sjögren's syndrome, but no LP-PCD. Postoperative examination did not reveal any findings suggestive of LP-PCD.

Eighteen cases of pulmonary CSH have been described in the English language peer-reviewed literature, including our case (Table 1) [4], [5], [6], [7], [8], [9], [10], [11], [12], [13], [14], [15], [16], [17]. Mean age is 62 years (range, 38–89 years). Cases included 7 men and 11 women. Fourteen patients had LP-PCD (marginal-zone lymphoma, n = 6; MGUS, n = 2; plasmacytoma, n = 2; MM, n = 1; lymphoplasmacytoid lymphoma, n = 1; gastric DLBCL, n = 1; pulmonary mucosa-associated lymphoma, n = 1), and 4 patients were without LP-PCD. In patients without LP-PCD, autoimmune diseases such as rheumatoid arthritis [16], antiphosphoid syndrome [14], and Sjögren's syndrome (as in our case) may be associated with CSH.

**Table 1:** Eighteen cases of pulmonary CSH have been described in the English language peer-reviewed literature, including our case.

Author	Year	Age	Sex	Associated conditions	Symptom	Maximum nodule size (mm)	Solitary or multiple nodules	Immunoglobulin type	Therapy
LP-PCD									
Kazzaz [4]	1992	60	M	Plasmacytoma	None	20	Multiple	κ	Lobectomy
Prasad [5]	1998	72	F	Lymphoplasmacytoid lymphoma	None	22	Solitary	IgM, κ	Segmentectomy
Yanyu [6]	2003	59	M	Extranoal marginal zone B-cell lymphoma	Chest and abdominal pain, weight loss	20	Multiple	Ig heavy chain rearrangement	Lobectomy
Papla [7]	2004	51	M	Multiple myeloma	Weight loss, fever	25	Solitary	IgG, κ	Lobectomy
Fairweather [8]	2006	69	F	Marginal-zone lymphoma	None	20	Solitary	B-cell clonality	Segmentectomy
Todd [9]	2010	75	F	MGUS	None	11	Solitary	NA	CT guided fine-needle aspiration
Ko [10]	2012	64	M	Marginal-zone lymphoma	NA	NA	Multiple	κ	Wedge resection
Rossi [11]	2013	54	F	MGUS	Dyspnea	NA	Solitary	NA	Wedge resection, steroids, azathioprine
Rossi	2013	89	F	Marginal-zone lymphoma	Fever	NA	Multiple	NA	steroids
Rossi	2013	50	F	Recurrent marginal-zone lymphoma	None	NA	Multiple	NA	chemotherapy
Rossi	2013	63	M	Plasmacytoma	Chest pain hemoptysis	NA	Solitary	NA	Lobectomy, chemotherapy
Kawano [12]	2013	80	M	Gastric DLBCL	NA	52	Solitary	Rearrangement of IgH	Wedge resection
Chen [13]	2013	54	F	Marginal-zone lymphoma of mucosa-associated lymphoid tissue	NA	19	Solitary	κ	Bilobectomy
Kokuho [14]	2017	38	F	Pulmonary mucosa-associated lymphoma, antiphosphoid syndrome	NA	42	Multiple	Immunoglobulin light chain	Surgical lung biopsy

Without LP-PCD									
Jones [15]	1996	54	F	Asbestos	None	30 (recurrence 20)	Solitary	Polyclonal	Wedge resection
Ionescu [16]	2005	50	F	Rheumatoid arthritis	None	20	Solitary	Polyclonal	Wedge resection
Lee [17]	2009	64	M	Asbestos	Cough, fever	45	Solitary	Polyclonal	VATS biopsy
Present case	2021	64	F	Sjögren’s syndrome	Cough	12	Solitary	Polyclonal	Segmentectomy

Pulmonary CSH was often asymptomatic, but sometimes symptomatic (chest pain, n = 2; cough, n = 2; fever, n = 2). Pulmonary nodules were solitary in 12 cases, and multiple in 6 cases. All patients without LP-PCD showed solitary nodules. Mean maximum nodule size was 25.7 mm (range, 11–52 mm). Therapy was bilobectomy in 1 case, lobectomy in 4 cases, segmentectomy in 3 cases, wedge resection in 5 cases, biopsy in 2 cases, CT-guided fine-needle aspiration in 1 case, administration of steroids in 2 cases, and chemotherapy in 2 cases. Therapy for all patients without LP-PCD was excisional resection of the lung. Treatment and prognosis of patients with CSH varied according to the defined pathology. CSH may precede the development of a LP-PCD, so underlying diseases should be followed-up thoroughly. Jones et al. reported the case of 54-year-old woman without LP-PCD who presented with a solitary asymptomatic focus of CSH in the lung and initially underwent lesion resection, but showed recurrence 10 years later [15]. Although pulmonary CSH is a benign tumor, long-term follow-up is warranted. Further accumulation of cases is necessary to clarify the optimal therapy and prognosis.

## 4 Conclusion

Pulmonary CSH is one differential diagnosis for pulmonary nodule enlargement in patients with autoimmune disease. Surgical resection appears to represent an effective therapeutic option for localized CSH, but long-term follow-up remains necessary.

## Consent

Written informed consent was obtained from the patient for publication of this case report and accompanying images. A copy of the written consent is available for review by the Editor-in-Chief of this journal on request.

## Provenance and peer review

Not commissioned, externally peer-reviewed.

## Ethical approval

No approval is required for this case report.

## Funding

No funding sources.

## Guarantor

Soichiro Kiya, MD.
Shigeyuki Morino, MD, PhD.
Keisuke Iwasaki, MD, PhD.
Akihiro Nakamura, MD, PhD.

## Research registration number

Not applicable.

## CRediT authorship contribution statement

Soichiro Kiya, MD: Surgeon performing the operation, writing of original article.
Shigeyuki Morino, MD, PhD: Revision of the manuscript.
Keisuke Iwasaki, MD, PhD: Pathologist diagnosed this case.
Akihiro Nakamura, MD, PhD: Revision and final approval of the manuscript.

## Declaration of competing interest

None.
